# Homœopathy: Its Tenets and Tendencies, Theoretical, Theological, and Therapeutical

**Published:** 1854-04

**Authors:** 


ART. XI. — *Homœopathy: its Tenets and Tendencies, Theoretical, Theological, and Therapeutical.* By JAMES Y. SIMPSON, M. D. Philadelphia: Lindsay & Blakiston.

The subject of homœopathy, in relation to its rise, progress, and the estimation in which it is held by the public, is well worthy the consideration of all members of the medical profession, whether it be regarded in the light of a strange fantasia or hallucination that at uncertain epochs seems to seize upon the human intellect, and affecting its powers of discriminating between truth and error, overturns the very foundations of correct reasoning, and for a time substitutes fallacies and delusions for well-established truths, and the vagaries of an enthusiast or the specious dogmas of an empiric for the careful deductions of philosophical inquiry, or whether it comes as a new element of competition in the daily routine of professional labor, having a tendency to depreciate the value of well-directed medical and surgical skill, and to deprive the industrious and honest laborer in the professional vineyard of the honor and emolument that he might reasonably expect to remunerate him for mental and bodily toil.

There is another consideration that appertains less to speculative inquiry or individual interests, but is still of importance as it affects the *esprit du corps* of a profession ever jealous of its honor.

There are physicians who tamper with homœopathy under the pretense of scientific investigation; and it is very necessary that their true position should be determined, both with reference to their intercourse with their professional brethren, and our organized societies and associations.

These persons, ostensibly acknowledging the principles of medical science, secretly pander to the prejudices that exist against legitimate practice, and coin the credulity of the deceived and deluded into a rich return for their treason to honor and truth.

Such considerations, necessarily briefly adverted to in this article, induced Professor Simpson, of Edinburgh, in the summer of 1851, to lay before the profession the first edition of a work entitled "Homœopathy, its Tenets and its Tendencies," the third edition of which has now been reprinted in Philadelphia by Lindsay & Blakiston. The immediate occasion of calling attention to this subject, as specified by the author in the preliminary remarks, chapter I, was to arouse medical men to a sense of their obligations to the profession, and to the societies of which they were members, and to warn them against the snares of a popular doctrine, and the avoidance of all tampering with their reputation, by secretly conniving at a system, that, by the most authoritative declarations of Hahnemann himself, was directly at variance with the principles and practice of legitimate medicine. It had already become necessary, in the course of this year, for the Royal College of Physicians and the Royal College of Surgeons of Edinburgh, the faculty and the physicians and surgeons of Glasgow, the Medical Society of London, and other medical associations, to prohibit their fellows and members from meeting, professionally, with those who compromised or abandoned their reputation from mercenary motives. The first edition was favorably received, and in two years it passed through three editions, clearly indicating the estimation in which the subject is regarded, and the merits of the work itself. In the present full and elaborate volume, the subject may be said to be fully examined in all its relations, and there is placed before the profession an interesting and able scrutiny into all the follies and cunning of the most pervading fraud upon the understandings of men that has ever been experienced or recorded. The delusions of particular localities, or ephemeral errors, we can readily understand, and await patiently the silent, but sure influence of time and truth to eradicate; but, in dealing with homœopathy, it seems to us a commendable effort to place before the public eye, and to arm the professional man with the necessary facts to contend against, the false statements, the perversion of facts, the illogical deductions and the unbounded extravagance of the claims of a system that has diffused itself and is cherished among people as different in mental constitutions as in language, among the rich and the poor, the ignorant and intelligent.

Without an effort this error cannot be reached and corrected. That kind of testimony which is necessary to enlighten the public mind, can alone be furnished by medical men, and they themselves should be well acquainted with the fallacies, contradictions and absurdities of the homœopathic art in order to instruct the ignorant, and enlighten the deluded believer in this stupendous imposition. Without attempting to furnish an analysis of the second and third chapters in their order of succession, we shall briefly call attention to their most importent contents, the points discussed in them, and the impropriety of permitting any physicians who tamper with homœopathy to remain in association with legitimate practitioners, on the ground that they do not advance, nor endeavor to advance, the interests of medical science, and therefore should be excluded; and the application of the standard of common sense to the dogma that the power of medicinal agents is increased by their attenuation. In the examination of this topic, ample numerical illustrations are afforded, whereby the most ignorant cannot fail to observe the extent of the absurdity in which he must be involved if he gives credence to such preposterous conception, and only equaled by the servile dotings of Hahnemann in his latter years, that a smell or inhalation of the higher potencies was quite as efficacious as the administration of the remedy itself.

Many honest, but ignorant, believers in homœopathy will declare that it succeeds as a mode of curing disease far better than allopathy, and with less subsequent disadvantage to the system. On this point Doctor Simpson furnishes various irrefutable proofs of the utter inefficiency of the system of practice when tested in hospitals, and relates, in proof of that self-deception which is so common when the mind is biased and inclined to believe in hoped-for results, an amusing anecdote of Doctor Henderson, Professor of General Pathology in the University of Edinburgh: "Some eight or ten years ago, an old schoolmate, having begun business as a druggist, in Liverpool, kindly sent Dr. S. a present of a small box of homœopathic medicines, —and a very beautiful painted box it was too. During the time it was in Dr. S.'s possession, he put it only to one use, viz., he gave it as an occasional plaything to his eldest son, who was then a child. The boy, reveling in his permitted amount of mischief, used in his sport to uncork the small bottles, empty their globules into a heap, and then refill the bottles from the general mass. Of course this had speedily the effect of altering and disarranging the contents of the entire lilliputian drug shop. The globules pertaining to the different bottles were more or less thoroughly mixed together, and sometimes, when the child was tired of his occupation, others at last filled the bottles from the general heap. A professional brother happening to call at Dr. S.'s house one day when Dr. S. was absent, saw the box and put it into his pocket. Many weeks after, the new proprietor of the box met Dr. S. and told him that he had been trying to practice homœopathically, at which Dr. S. expressed his regret, and he added that he had seen some wonderful effects and cures from using the drugs contained in Dr. S.'s own former homœopathic box! Wrongly, as Dr. S. thinks now, he did not at the time tell this physician that the globules were elaborately commixed." The result was, that the professor became a convert to homœopathy by a process that no one will undertake to say was one of common sense or philosophy, to the great mortification of his colleagues and to the injury of medical science. The remedial powers of nature in surmounting and curing diseases, are necessarily unknown to the public, even to that portion of the community that may be pronounced educated and enlightened; they are, therefore, not relied upon for relief when disease attacks the frame. If homœopathy is then appealed to, lends its aid, and the result is propitious, the wonder arises that so much good is wrought out of apparently such inadequate means.

Fortunately for the interests of science, satisfactory tests have been furnished to prove the inefficiency, worse than that, the pernicious effects of the practice of homœopathy in the hospitals of Liverpool and others, which will be referred to hereafter. "The house physician of the hospital above-mentioned, having become convinced of the nullity and danger of homœopathy, gave up his appointment aud published an exposition of the system pursued (on the nothingness of homœopathy) with an account of cases which clearly shows that the so-called cures were recovering from ordinary ailments by the efforts of nature, which were frequently a long time under treatment. Whereas, by a proper medication and attention at the onset, they might probably have been recovered in a few days, and that many of the more serious cases got worse instead of better for the want of active treatment."

A report by Doctor George Fleming, of Dundee, sojourning at Vienna six months, and attending the Homœopathic Hospital there, confirms the preceding statement. At another and older hospital at Gumpendorp, in the suburbs of Vienna, there had ceased to be any attendance of pupils or patients, for the loss of confidence and success. It is also stated, on the authority of Doctor Gerson, who has studied in various schools, and has lately come on a visit to Edinburgh, that during the last six or eight years neither the medical profession, nor the public of Germany, have now confidence in homœopathy; it is tolerated because it is conceived to be a very harmless and innocent species of quackery. Such is the uniform testimony from other sources entitled to the highest consideration for veracity and correctness. Having been publicly tested in Germany, although as a theory well adapted to the imaginative mental temperament of the German people, homœopathy must gradually die in its place of birth. It has been weighed by the good sense and experience of its friends and admirers, but they have come to the irresistible conclusion that it is inert and powerless in acute diseases, and from its very negation of active treatment, must fail as a means of relief when most speedily required. Notices of some other public and hospital experiments have been published. A German Homœopathist, practicing in Russia, was invested, by the Grand Duke Michael, with full powers to prove by comparison of facts the advantages of homœopathic measures over ordinary treatment, and a certain number of patients in a military hospital were intrusted to his care. At the expiration of two months he was not permitted to proceed any further. For in comparing results, it was seen that within this period out of 457 patients treated by the ordinary means, 364 or three-fourths were cured, and none died; whereas by the homœopathic method tried on 128 patients, one-half were cured and five died. The Russian government also tried, in two hospitals, the comparative treatment of a number of patients with homœopathic globules, and a number of other patients with no drugs of any kind, and the results were found similar in both instances. A committee appointed by royal order, at Naples, to superintend the treatment of a number of hospital patients by homœopathy, reported as the result of their observations:

1st. That the homœopathic treatment produced no effect.

2d. That it had the serious inconvenience, in several of the patients, of preventing the employment of remedies by which they might be cured.

In another instance, after a most careful and candid observation of the effects of remedies in the London Homœopathic Hospital, a medical gentleman, whose character for veracity is endorsed by Professor Simpson, came to the conclusion "that as to practice, homœopathy is truly a nonentity, and literally the swallowing of names only." We will add one more of the practical tests which are desired by those who, from want of opportunity, are obliged to rest their opinions on the observations of others; it was furnished by M. Andral in one of the Parisian Hospitals, when the claims of the system were brought before the French Academy of Medicine: "the result was a failure; he tried it in 130 or 140 patients, in the presence of the homœopathists themselves, adopting every requisite care and precaution, yet in no one instance was it successful." In his report to the academy, he says: "That not only did quinine produce no ague, aconite no plethora, sulphur no itch, arnica no pains as if from contusion, but in every instance of disease he was obliged to resort to allopathy, as, under the homœopathic mode of treatment, the symptoms went on from bad to worse."

The above are taken from a mass of facts adduced by Professor Simpson to convince the ignorant and deluded among unprofessional men, of the absurdity of the claims of the so-called homœopathic system to their confidence. The refutation of the pretensions, as a curative method, has been abundantly established by the above public experiments and tests. They were not done in a corner, but they were executed under conditions and circumstances furnished and approved by the homœopathists themselves. In London, Leipsic, Vienna, Russia, France and Naples, in public institutions, and before the eyes of the community, these experiments were conducted, where no individual nor professional interests were permitted to modify or to pervert the results of the investigations. The personal experiments of Andral, and the daily experience of professional life, sufficiently confirm the fallacy of the declaration, that quinine will cause ague, or sulphur the itch; or that the imaginary properties of remedial agents will cause objective phenomena in accordance with the transcendental vagaries of the spiritualizing votaries of an illusory creed. For a period of twelve years, Hahnemann states, he was tracing the origin of an incredibly large number of diseases to their true source, the itch. When this is seriously propounded by him for belief, it would seem that the most bigoted and credulous of his dupes must shrink from such amazing folly as to believe that nervous debility, hysteria, hypochondriasis, mania, imbecility, madness, epilepsy and convulsions of all sorts, rachitis, scoliosis and cyphosis, caries, cancer, fungus hæmatodes, malignant organic growths, gout, hæmorrhoids, jaundice, cyanosis, dropsy, amenorrhœa, hæmorrhage from the stomach, nose, lungs, bladder and womb, asthma and ulceration of the lungs, impotence and barrenness, megrim, deafness, cataract, amaurosis, urinary calculus, paralysis, defects of the senses and pains of the various kinds that figure in systematic works on pathology as peculiar and independent diseases, all arise from itch; and yet we find before us in this volume, the evidence that men of intelligence give credence to it, and a learned theologian couples this doctrine with the divine command to heal the lepers. Can delusion go farther? An attempt is made by Hanhemann to prove the infallibility of the so-styled universal law by the cure of small-pox by cow-pox, and extends it to other connate diseases. In the experiments of Doctor Willan we have an unimpeachable witness, for he is summoned on the stand by the great lawgiver himself, who asserts "that small-pox coming on after vaccination, as well on account of its greater strength as its great similarity, immediately removes entirely the cow-pox homœopathically, and does not permit it to come to maturity, as Willan on Vaccination, and many others testify."

To quote from Dr. Simpson in reply, and in refutation of this untenable statement, it has been shown by Dr. Willan as early as 1800: "That when a person was inoculated and vaccinated at the same time, both proved effective, for the vaccine vesicle proceeded to its acme in the usual number of days, and the variolous disease was attended with a pustular eruption on the skin. Dr. Willan gives a drawing and notice of a case of the conjunction of small-pox and cow-pox upon the same individual, which is peculiarly interesting as bearing upon the homœopathic law. He represents in his work, Plate I, Fig. 3, the arm of a boy who had been inoculated with variolous matter and vaccine matter. The vaccine vesicle progressed and is represented of full size, and *within* the border of one side of the vaccine vesicle is a small-pox pustule which rose and maturated in that position. Dr. Willan further states that matter taken from this inclosed pustule produced small-pox, while the lymph from the vaccine vesicle communicated the cow-pox." M. Legendre has collected more than fifty, and Clerault more than one hundred cases of the simultaneous appearance of these two diseases, and in an epidemic at Marseilles, in 1828, sixteen persons died, all affected with co-existent cow-pox and small-pox. From the domain of connatural or allied diseases, Hahnemann also adduces, as another instance of the truth of the universal law, the homœopathic cure and the preclusion of hooping-cough by the measles. Without attempting to controvert the well-known coincident prevalence of the two diseases, and the frequent succession of the one upon subsidence of the other, to an extent so marked and frequent as to lead to the opinion, by many distinguished pathologists, as Copland, Desmelles, and others, that the one predisposes to an attack of the other, he again perverts the language of Bonsquillan, and with the same disregard for truth as in the case cited above of Dr. Willan. Dr. William Thompson, Professor of the Practice of Physic in Glasgow, has translated an extract from Bonsquillan's French edition of Cullen's Practice, in which he expresses his belief that so far from having preventive power "there is much reason to believe that measles prepares the way for hooping-cough, and that that preparatory influence is exercised through the mucous glands."

Well may Dr. Simpson, with such evidence before him of the duplicity of this self-styled high-priest of nature, exclaim, "They drew aside the vail, and where they expected a mystery they discovered a fraud."

We furnish, from copious stores of similar facts, another instance of the total failure in its application of the universal law in the eradication of miliaria by the curative substitution of measles, as stated in the language of Hahnemann: "A chronic herpetic eruption was entirely and homœopathically cured by the breaking out of the measles, as Kortum observed. An excessively burning miliary rash on the face, neck, and arms, that had lasted six years, and was aggravated by every change of the weather, under the influence of the measles assumed the form of a swelling on the surface of the skin; after the measles had run its course, the rash was cured and returned no more." The fallacy in the application of the rule in this instance must be obvious to any, the most unreflecting. The one disease, of an epidemic character, spreads by atmospheric and contagious influences, having a determinate course, affecting all the fluids and tissues of the economy, and bearing no more similarity to miliaria, a localized disease, than, in the language of Dr. Simpson, a salmon does to a leopard because both have spots on the surface. The other analogous diseases adduced by Hahnemann are equally inadequate to sustain the doctrine that like cures like; but as his errors become more patent his assertions become stronger." After telling us that his object is now to speak of something "determined and indubitable," he proceeds to state that the small-pox upon the principle "similia similibus curantur," has, as a curative agent, "removed and cured a number of affections with similar symptoms," among which are amaurosis, chronic opthalmia, deafness, dyspnœa, testicular swelling, and a dysenteric state of the bowels. In answer to this argument, the records of medicine fail to show that amaurosis and testicular swelling are the sequelæ of small-pox, or are cured by an attack of it; "that chronic ophthalmia and dysentery may be caused by or in concomitant symptoms, is no evidence that they are necessarily cured, homœopathically, by it; but admitting that there may be some remote connection between the pathological condition of the cause and the sequence, the law is invalidated upon his own declaration, that the remedies for the disease must be not only similar, but they must be the most similar possible, or that there must be the most accurate similarity." Can absurd pretense go farther, or to any unprejudiced mind is it necessary to urge facts to prove the wide variance between a chronic conjunctivitis, or dysentery and small-pox? When an advocate of a weak cause is driven to such props, he has saved his antagonist trouble, by reducing his own proposition to the extremest absurdity. Chapter XVII is devoted to those points which involve the purposes and objects of employing remedial agents, and as a practical art, for the relief of mankind from all the curable ills to which flesh is heir, the importance of the subject is ably and fully canvassed in this one of the most interesting in this very interesting and instructive book. Here is developed the entire disregard of Hahnemann and his disciples for pathological observations, conditions and results: they are pronounced by him as illusory, the vagaries of a dream, utterly unreliable and as leading astray. He does not allow, as a condition of disease, an internal change of texture or function evidenced by external signs: the latter only, when rightly interpreted, the exponents of change, in function, or organic structure. He declares that the symptoms alone are worthy of attention. "Every thing," he observes, "of a really morbid character, and which ought to be cured, consists solely in the sum total of the symptoms, by means of which the disease demands the medicine requisite for its relief, while, on the other hand, every internal cause, every occult quality or imaginary morbific principle, is nothing but an empty dream." He also adds: "That besides the collective symptoms, nothing can be discovered in any way in diseases, wherewith they could express their need of aid; it undeniably follows that the sum of all the symptoms in each individual case of disease must be the sole indication, the sole guide to direct us in the choice of a curate remedy." It would be adding unnecessarily to the length of a mere synopsis to pursue any further the abundant proofs furnished by the author upon this subject, or to add an argument in favor of the indispensable necessity of a thorough knowledge and cultivation of pathology as the groundwork of legitimate medicine. Enough has been extracted from the pages before us to excite interest, we hope, for a further examination (in the work itself) of the thorough antagonism and contrariety between allopathy and homœopathy, on this most important subject. As it has not been convenient to adhere strictly to the arrangement of the work itself in the imperfect manner in which an analysis has been attempted, we shall pass over many other branches, treated of in the remaining chapters in the same clear and satisfactory manner, apparent in the arrangement of facts and arguments that characterize the former ones. Dr. Simpson furnishes abundant evidence that not alone in this country is homœopathy seized upon by the mercenary or unsuccessful licentiates of legitimate medicine, who, unscrupulous as the founder of the system, prey upon the dupes of their impostures. It is well known that Hahnemann failed of success in the practice of medicine, and then turned his attention to more facile modes of money making than the toilsome paths of rectitude and true science. Long before 1810, the year of the publication of his Organon, he had disgraced himself and lost his position as a professional man, by vending a nostrum under the name of Pnœum, that consisted of nothing but borax. Having once thrown aside all regard for professional respect, he exerted all his efforts to establish a new system, which he well knew would, from its very extravagance, arrest the attention of the curious, and impose upon the credulous and impulsive with its dynamizations, and potencies, and spiritualizations. He struck a vein in the mental constitution that as long as there is a living man or woman to be moved by wonder, or tempted by curiosity, to adventure upon new paths of science or pleasure, will never be exhausted.

It is curious to note from the pens of reliable authority, the latter days of Hahnemann in Paris, where his celebrity ceased not to attract crowds, even when life and intelligence were on the wane, nay, almost extinct. His young wife prescribed to hundreds of invalids from all parts of the world; some attracted by curiosity, some with the vain hope of deriving benefit. His crowded salons bore witness to the notoriety he had attained, and while she, who is described as being pretty and intelligent, recorded the symptoms and prescribed the remedies, the aged founder of homœopathy, in senile torpor, reposed in a well-stuffed easy-chair, mechanically puffing his Meerschaum, and gazed with lusterless eyes upon those who were around him.

Although there are many homœopathic practitioners in Western New

York, and the doctrine itself has been popular, yet to the credit of the profession it must be said, that very few regular members have tampered with it. The incentives to do so have been very strong; urgent appeals have been made by the popular voice to the dejected, plodding, and dispirited practitioner to abandon his obsolete and unpopular system, and become the advocate and follower of the new and favorite doctrine. Golden harvests have been promised, and the success that has attended more dashing and unscrupulous competitors, has been obvious. We cannot but admire the abiding influence of those moral precepts, that, infused into the mind of the student while pursuing his preparatory course, protect him in after life while struggling with obscurity, perhaps penury, in a toilsome and anxious profession, from swerving from the path of rectitude and honor.

Though we possess many works on the subject of homœopathy, and among them those of Dr. Hooker are especially agreeable, Dr. Simpson's volume will amply repay a perusal, and we can safely recommend it to our professional brethren. W.

